# Supplementary material for: Predictive coding in musical anhedonia: A study of groove
Source: PLoS One. 2024 Apr 23;19(4):e0301478. doi: 10.1371/journal.pone.0301478 (PMC11037533; doi:10.1371/journal.pone.0301478)
Supplement: S1 File — (DOCX) [file pone.0301478.s001.docx]

# Supplementary Materials

For **Predictive Coding in Musical Anhedonia: A Study of Groove**

by Peter Benson, Nicholas Kathios, Psyche Loui

# Supplemental Methods

| Song Title | Artist | Perceived  Complexity | Total  Onsets | Duration  (s) | Loudness  (RMS) | Initial Tempo  (BPM) | Syncopation | Estimated  Complexity | Complexity  Tertile |
| --- | --- | --- | --- | --- | --- | --- | --- | --- | --- |
| A Kind Of Magic | Queen | 0.4 | 58 | 8.802 | -24.14 | 130.13 | 16 | 0.728 | Low |
| (Sittin' On) The  Dock Of The Bay | Otis Redding | 0.40777816 | 54 | 10.8 | -27.58 | 103.69 | 52 | 0.653 | Low |
| Smells Like  Teen Spirit | Nirvana | 0.47575974 | 58 | 9.788 | -24.07 | 116.32 | 64 | 0.444 | Low |
| Boogie  Wonderland | Earth, Wind,  & Fire | 0.57295016 | 57 | 8.84 | -24.01 | 131.25 | 16 | 0.947 | Low |
| Vultures | John Mayer | 0.78435927 | 52 | 11.41 | -24.34 | 97.51 | 28 | 0.611 | Low |
| Discipline | Nine Inch Nails | 2.0911516 | 62 | 9.378 | -22.76 | 122.01 | 16 | 1.744 | Intermediate |
| Pass The Peas | The J.B.'s | 2.15064731 | 58 | 11.31 | -28.44 | 99.71 | 136 | 1.578 | Intermediate |
| The Pump | Jeff Beck | 2.18884353 | 58 | 15.975 | -21.4 | 86.8 | 33 | 2.809 | Intermediate |
| Roxanne | The Police | 2.21600864 | 62 | 11.821 | -22.63 | 136.32 | 45 | 1.74 | Intermediate |
| Dreamin' | Loleatta  Holloway | 2.41311954 | 65 | 12.997 | -28.23 | 118.35 | 15 | 1.84 | Intermediate |
| Alone + Easy  Target | Foo Fighters | 3.13038202 | 47 | 12.109 | -23.14 | 133.13 | 128 | 2.356 | High |
| Diggin' On  James Brown | Tower Of  Power | 3.31385354 | 45 | 9.236 | -24.51 | 128.69 | 64 | 2.975 | High |
| Cold Sweat | James Brown | 3.76275565 | 60 | 10.148 | -27.16 | 111.66 | 96 | 2.824 | High |
| Hyperpower | Nine Inch Nails | 3.90190277 | 85 | 16.262 | -19.66 | 86 | 88 | 4.027 | High |
| Rock Steady | Aretha Franklin | 4.393996 | 92 | 13.964 | -22.6 | 104.27 | 202 | 4.62 | High |

**Table S1:** Chosen stimuli from the Senn et al. (2023) corpus for the present study, along with measures of acoustic and perceptual features of these stimuli. Song titles, artists, perceived complexity, total onsets, duration, loudness, and tempo values are taken directly from Senn et al. (2023). Syncopation values come directly from the Lucerne Groove Research Library (<https://www.grooveresearch.ch/index.php?about>), and estimated complexity values are from Senn (2023).

| **Acoustic/Perceptual Feature** | **Low Complexity Tertile n=5**  M (SD) | **Intermediate Complexity Tertile n=5**  M (SD) | **High Complexity Tertile n=5**  M (SD) |
| --- | --- | --- | --- |
| Loudness | -24.83 (1.5) | -24.69 (3.4) | -23.41 (2.7) |
| Total Onsets | 55.80 (2.7) | 61 (3) | 65.80 (21.6) |
| Tempo | 115.78 (15.2) | 112.64 (19.5) | 112.75 (19.1) |
| Duration | 9.93 (1.2) | 12.30 (2.4) | 12.34 (2.9) |
| Perceived Complexity*** | 0.53 (0.2) | 2.21 (0.1) | 3.7 (0.5) |
| Estimated Complexity*** | 0.68 (0.18) | 1.94 (0.49) | 3.36 (0.93) |
| Syncopation Index* | 35.2 (21.8) | 49 (50.21) | 115.6 (53.45) |

**Table S2:** Mean and standard deviation for each acoustic & perceptual feature across the three stimulus tertile complexity groups. Asterisks indicate significant differences across groups as a function of a one-way ANOVA. * *p* < 0.05; *** *p* < 0.001

# Supplemental Results


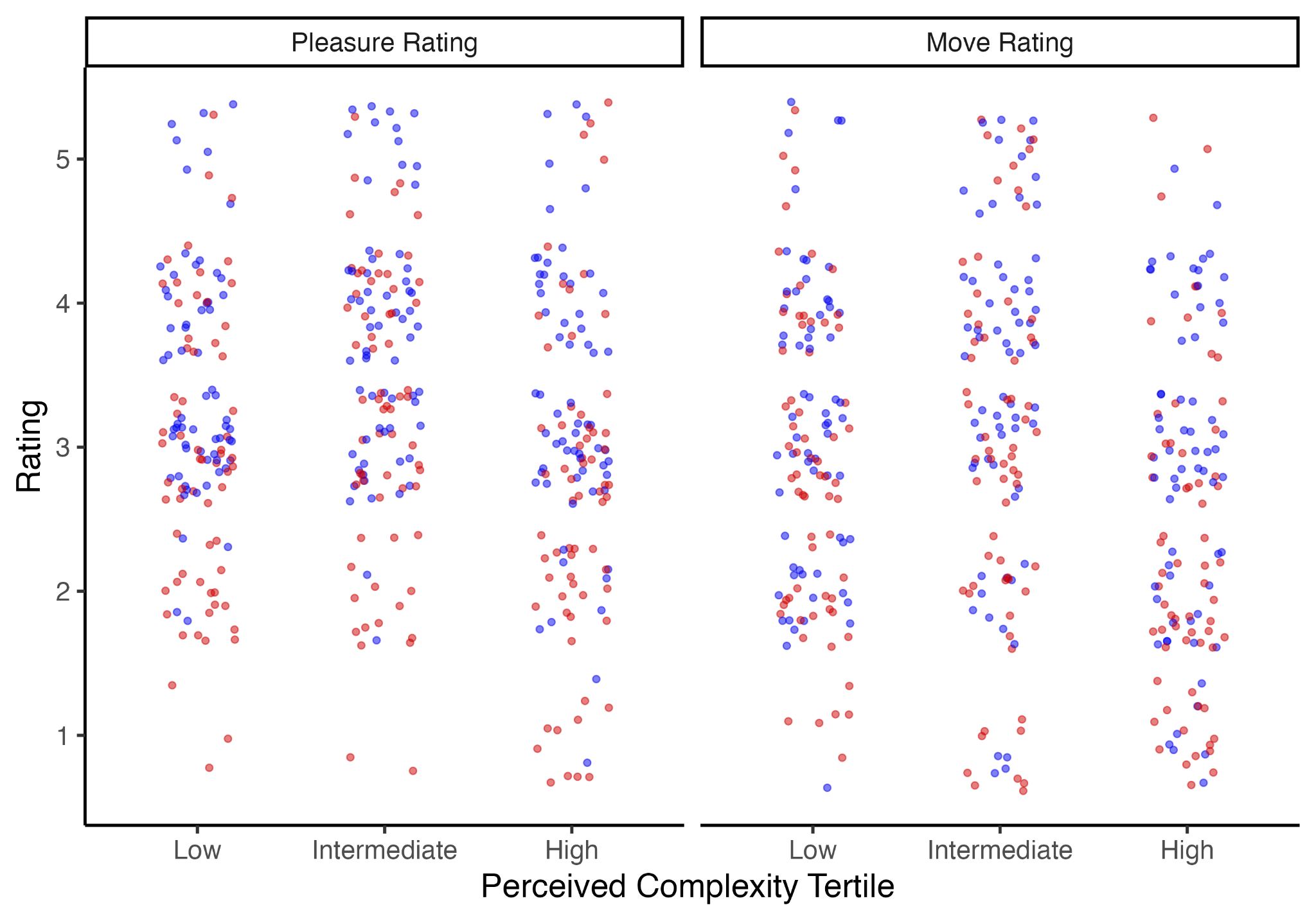

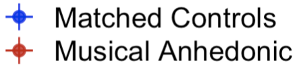


**Supplemental Figure 1:** Raw pleasure and move ratings across the perceived complexity tertiles for musical anhedonics (red) and matched controls (blue).

*Musical Training & Groove*

A linear mixed-model relating musical training on the Gold-MSI to rating with an interaction term of complexity tertile found no significant relationship for either rating type, stimulus complexity tertile, or an interaction between the two. See Supplemental Table S3 for model statistics.

| **A) Pleasure Ratings** | | |
| --- | --- | --- |
| **Model Term** | *F*  *(numerator df, denominator df)* | *p* |
| Gold - MSI Musical Training | 0.1 (1, 216) | 0.76 |
| Stimulus Tertile Complexity | 2.57 (2, 14) | 0.2 |
| Gold - MSI Musical Training X  Stimulus Tertile Complexity | 1.51 (2, 281) | 0.22 |
| **B) Wanting to Move Ratings** | | |
| **Model Term** | *F*  *(numerator df, denominator df)* | *p* |
| Gold - MSI Musical Training | 0.35 (1, 216) | 0.56 |
| Stimulus Tertile Complexity | 0.95 (2, 12) | 0.41 |
| Gold - MSI Musical Training X  Stimulus Tertile Complexity | 0.67 (2, 297) | 0.51 |

**Table S3:** Model results treating pleasure (**A**) and move (**B**) as a function of Gold - MSI musical training scores, with an interaction term for stimulus tertile complexity group.
